# FIA automatic dilution system for the determination of metallic cations in waters by atomic absorption and flame emission spectrometry

**DOI:** 10.1155/S146392469600003X

**Published:** 1996

**Authors:** Jorge M. P. J. Garrido, Rui A. S. Lapa, José L. F. C. Lima, Cristina Delerue-Matos, João L. M. Santos

**Affiliations:** CEQUP/Departamento de Química: Fīsica Faculdade de Farmácia Universidade do Porto Rua Aníbal Cunha, 164 Porto 4050 Portugal


Journal of Automatic Chemistry, Vol. 18, No. 1 (January–February 1996), pp. 17–21

### FIA automatic dilution system for the determination of metallic cations in waters by atomic absorption and flame emission spectrometry

**Jorge M. P. J. Garrido, Rui A. S. Lapa*, José L. F. C. Lima, Cristina Delerue-Matos and João L. M. Santos**

*CEQUP/Departamento de Química: Física, Faculdade de Farmácia, Universidade do Porto Rua Aníbal Cunha, 164, 4050 Porto, Portugal*

*A fully automated flow injection analysis (FIA) dilution system based on the zone sampling technique is described. The system relies on the use of a PC-compatible microcomputer for instrumental control and data acquisition and processing. The computer controls two rotatory valves which select different portions of the injected sample plug and then are re-sampled toward the detector. The system automatically chooses a suitable dilution factor for each sample. The dilution system was coupled to an atomic absorption spectrometer and to a flame photometer for the determination of Ca, Mg, Na, K, Cu, Zn and Fe in highly concentrated water samples. The methodology affords up to 10 000-fold dilution factors with good precision (RSD < 3%) and high sampling rates (never lower than 90 determinations per hour).*

### Introduction

Sample handling is frequently a limiting step in conventional analytical procedures. Manual pre-treatment of the samples, like multi-step dilutions, could become a tedious time-consuming operation. Well specified and designed automatic systems could improve the efficiency of the analysis both in the quality of the measurements and the value of the results.

Flow injection analysis (FIA) is a simple, low cost and versatile methodology that has proven to be a very useful tool for automating various steps of analytical procedures including the adjustment of the specie's concentration to the detector's specifications. Dilution chambers [1–3], stream splitting [4], dialysis [5] and the injection of microlitre-volume samples [6] are FIA techniques that could be used to obtain on-line dilutions. These and other procedures for dilution have been reviewed by Fang [7], Tyson [8] and more recently by Luque de Castro [9]. The dilution yielded by these techniques is usually dependent on the manifold design and identical for all the samples apart from their concentration. Consequently, the dispersion coefficients achieved, although appropriate for the more concentrated samples, could be too high for the lesser concentrated ones, which would compromise the sensitivity and the detection limit of the analysis. Moreover, some of these techniques have limited sampling rates.

Zone sampling [10] is a versatile FIA technique which combines the possibility of determining a wide range of concentrations, yielding high dispersion coefficients, with high sampling rates. However, its manual operation (which is dependent on a precise timing control of the zone sampling process) is limited in terms of reproducibility. Moreover, to take full advantage of the zone sampling capacities, in terms of increased sample throughput, calibration facility and dilution efficiency, an automated system is required. In water analysis samples often have a wide range of concentrations which require different dilution levels that are difficult to predict. The FIA system described in this paper automatically selects, by means of the evaluation of the analytical signal obtained from the sample measurement, a suitable dilution factor for each sample according to a pre-set calibration procedure. This system, coupled to an atomic absorption spectrometer or a flame photometer, was used in routine analysis of natural and concentrated water samples for the determination of high levels of metallic cations.

### Experimental

### *Hardware*

The determinations of Ca, Mg, Zn, Fe and Cu were carried out using a Perkin-Elmer Model 5000 atomic absorption spectrometer; a Jencons Model PFP7 flame photometer using a butane flame was used for Na and K. A Gilson Model 221 was used as an autosampler.

An 486DX-based microcomputer was used as control unit. Communication between the computer and the analytical system was through a data acquisition and control interface PC-LABCard model PCL-812PG with an A/D 12 bit converter, from Advantech; final reports were printed by an LX 800 Epson printer.

### *Flow injection manifolds*

For the propulsion of the solutions, Gilson Model Minipuls 3 peristaltic pumps and Tygon pumping tubes of the same brand were used. Tubing was made of Omnifit PTFE ($\phi$ = 0·8 mm) with Gilson end-fittings and connectors, home-made Y-joints (used as confluence points), and pulse dampers constructed of perspex [11]. For sample injection and for the re-sampling process, two Rheodyne Model 5020 injection valves were used. These valves were equipped with an electric motor, micro-switches and an interface for microcomputer control (figure 1).

* *Correspondence to Dr Lapa.*

0142-0453/96 $12.00 © 1996 Taylor & Francis Ltd.

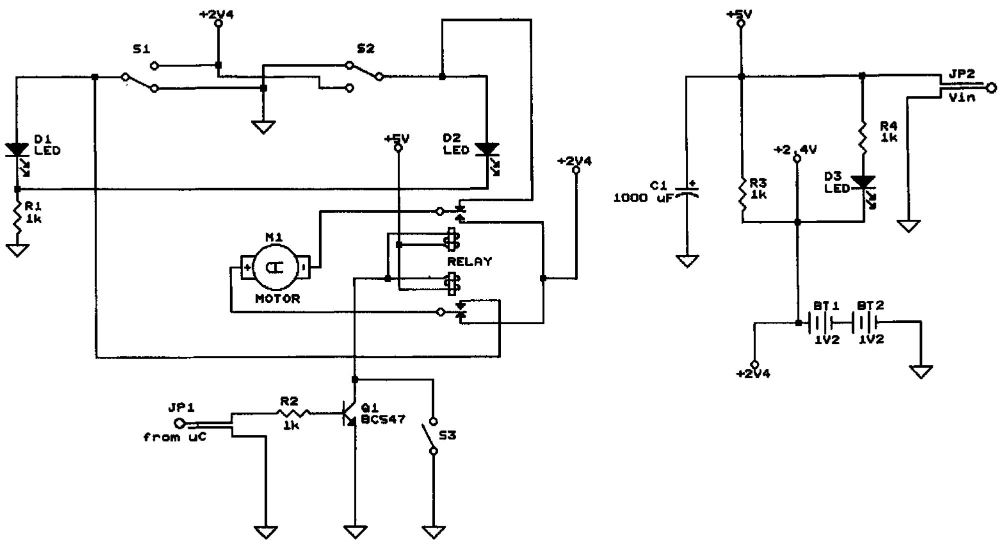

*Figure 1. Schematic diagram of the electric motor circuit used for rotation of the valves.*

*Software*

The software used to run the system (figure 2), in Microsoft QuickBASIC 4.5, allowed the computer to control the peristaltic pumps, injection valves, sampler and data acquisition during processing. The software also managed calibration data, concentration calculations and data storage. Subroutines ensured recording of the baseline, injection of the samples and standards, recording of the response signal and its evaluation in terms of peak height [12]. Advantech PCL-812PG card routines were used for data acquisition and linked to the software. The data collected were processed by using a least squares regression.

The Gilson autosampler was controlled by the microcomputer via TTL (Transistor–Transistor Logic) signals. The peristaltic pumps were controlled by the microcomputer via two TTL signals for start/stop and direction control and via an analogic signal for speed control.

*Reagents and solutions*

Chemicals were all of analytical grade. Ca, Mg, K and Na standard solutions used in the establishment of the calibration curves were prepared by dilution of BDH Spectrosol 1000 mg/l standard solutions. Cu and Zn standard solutions, due to the highly concentrated standards required, were prepared from $Cu(NO_3)_2 \cdot 3H_2O$ and $Zn(NO_3)_2 \cdot 4H_2O$ from Merck. Fe standard solutions were prepared from iron wire from BDH.

*Zone sampling procedure*

The zone sampling technique relies on the cleavage of an aliquot of the injected sample, which is directed toward the detector. In the developed manifold (figure 3), the sample is injected by an injection valve ($V_1$) into a first carrier stream ($Q_2$). Following the dilution coil ($R_1$), where it endures a low dispersion, the sample goes into the loop of a second valve ($V_2$) before flowing to waste. After the front portion of the dispersed sample has been discharged, $V_2$ is rotated and an aliquot of the trailing edge of the sample plug is cleavaged, re-sampled into a

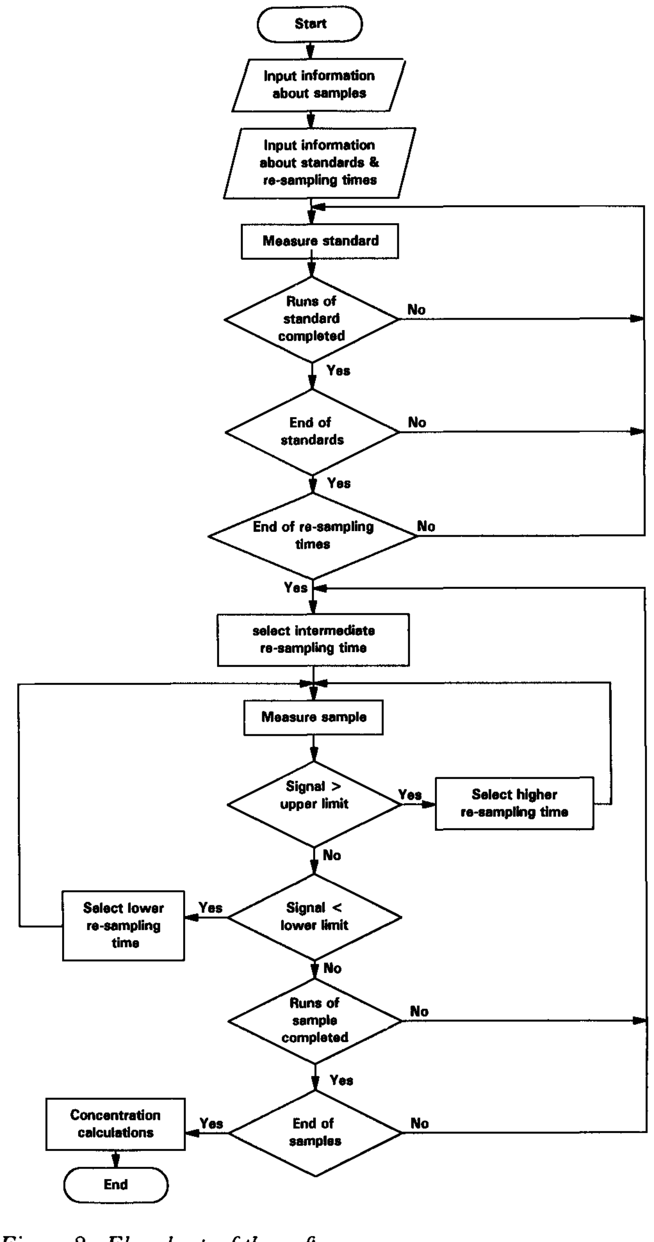

*Figure 2. Flowchart of the software.*

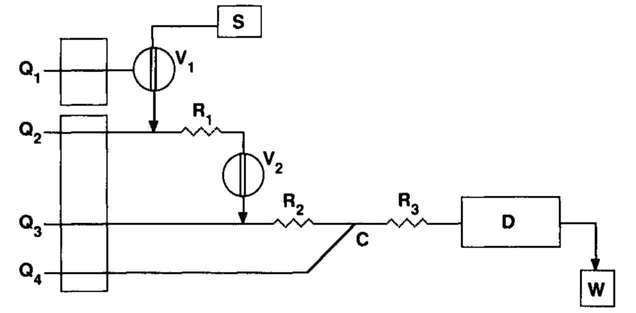

*Figure 3. Zone sampling manifold used for the determination of metallic cations in waters. S: Sample; D: Detector; W: Waste; Flow rate: $Q_1$ = 7·0 ml/min, $Q_2$, $Q_3$ and $Q_4$ = 4·8 ml/min; Coil length: $R_1$ = 180 cm, $R_2$ = 33 cm and $R_3$ = 64 cm; $V_1$ and $V_2$: Injection valves (loop length—$V_1$ = 16 cm and $V_2$ = 70 cm); C: confluence point.*

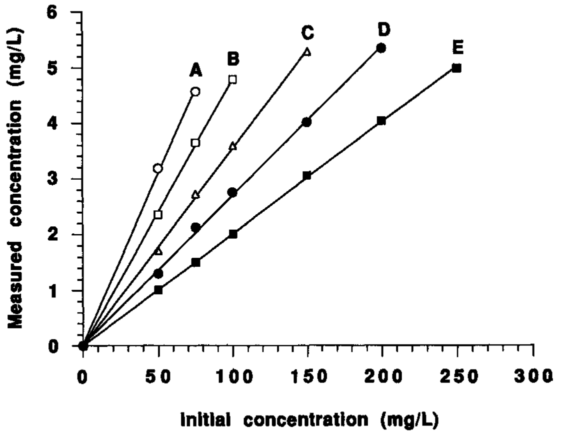

*Figure 4. Relationship between initial concentration and measured concentration of 50, 75, 100, 150, 200 and 250 mg/l calcium standards analysed at different re-sampling times: A: 20 s; B: 21 s; C: 22 s; D: 23 s; E: 24 s.*

second carrier stream ($Q_3$) and directed toward the detector. The re-sampling time ($t_r$) elapsed between injection and cleavage, defined the portion of the dispersed sample detected: if $t_r$ is increased the sample aliquot re-sampled decreases.

*Reference procedures*

Results were compared with the reference methods recommended by ASTM (American Standard for Testing and Materials) [13]: Ca, Mg, Cu, Zn and Fe were determined by Atomic Absorption Spectrometry (AAS) and Na and K by Flame Photometry (FP).

### Results and discussion

In process control or routine analyses, the concentration of the samples to be assayed is frequently distributed over wide ranges, particularly in water analysis. So samples often have to be diluted and the most appropriate dilution factor calculated. Of the various FIA techniques that could be used for automating dilution processes, zone sampling is attractive because the dilution factor can be continuously and promptly altered, by changing the re-sampling time, so it is unnecessary to modify the manifold configuration. However, to guarantee the quality of the analysis, the re-sampling time and consequently rotation of the valves must be capable of precise reproduction. By doing so, a linear relationship between the initial concentration and the measured concentration is obtained (figure 4).

Moreover, by automating the control of the valves, reproducibility was greatly enhanced—a standard deviation of 1·8% was obtained with 60 successive injections of a 10 mg/l calcium standard at a re-sampling time of 15 s.

When using zone sampling a calibration curve had to be constructed for each re-sampling time ($t_r$), which involves preparing appropriate standards and running them at the established $t_r$. During analysis, the dilution factor obtained for the standards at this $t_r$ will be reproduced for all the samples regardless of their concentration. However,

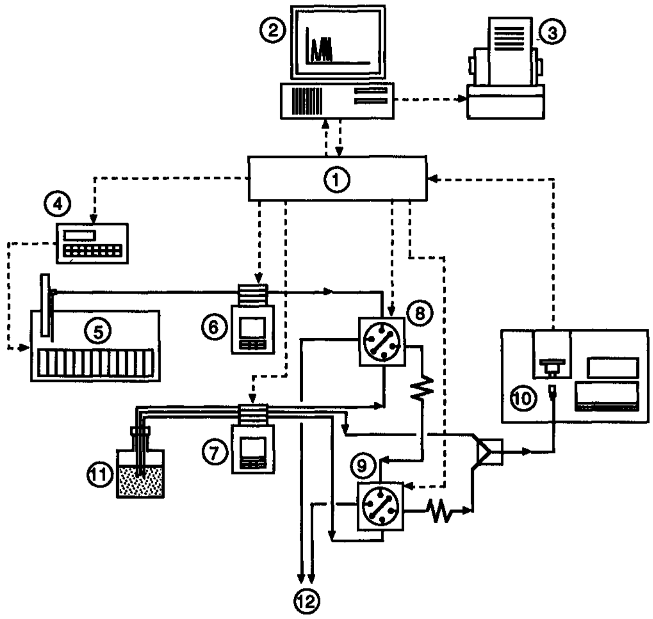

*Figure 5. Automatic dilution system. 1. Data acquisition and control interface card; 2: Microcomputer; 3: Printer; 4: Sampler control unit; 5: Sampler; 6: Peristaltic pump—$P_1$; 7: Peristaltic pump—$P_2$; 8: Injection valve—$V_1$; 9: Injection valve—$V_2$; 10: Detector; 11: Carrier solution; 12: Waste.*

although the pre-set $t_r$ would probably be adequate for the majority of the samples, its characteristic dilution factor would not be appropriate for samples with higher than expected concentrations. On the other hand, if a sample was less concentrated, then the same $t_r$ could yield an overdilution that would decrease the sensitivity and the detection limit of the analysis. In both cases it would be necessary to construct new calibration curves with different re-sampling times and with new standards.

In the system described here (figure 5), the calibration step will cover a wide concentration range and will enable the system to select the most convenient dilution factor for each sample. This calibration step involves the preparation of two or three calibration curves using an identical number of re-sampling times and of sets of standards, each covering a particular concentration range. Consequently, the dilution factor that will allow the analysis of the samples within the lower range of concentrations will be defined by the lower $t_r$ and the lesser concentrated standards set. The higher $t_r$ will be used with the higher concentrated standards, establishing the upper concentration limit.

During analysis, the system uses as first dilution factor the one corresponding to the intermediary $t_r$; if the analytical signal is lower than the signal obtained with the less concentrated standard used for the calibration curve, the system will repeat the analysis of the sample at the lower $t_r$. However, if the signal is higher than the one obtained with the more concentrated standard, the analysis will be repeated at the higher $t_r$.

In order to obtain a high sample throughput, the flow rate of $Q_1$ was set to 7·0 ml/min because it was necessary to fill the loop of the first valve ($V_1$) immediately after the system had decided whether a new sample would be analysed or the analysis of the sample would be repeated at a different $t_r$. The peristaltic pump responsible for the sample's introduction ($P_1$) rotated only briefly and was stopped during the remainder of the analysis.

The re-sampling of an aliquot of the trailing edge of the sample plug, rather than its front portion, is justified by the lower concentration profile of the former, due to a higher dispersion which gives rise to smaller fluctuations in the cleavage process [10]. Special attention was paid to the flow rate of the carrier solution ($Q_2$), the length of the coil ($R_1$) and the volume of the sample injected in $V_1$ for the same reason—they influence the dispersion of the sample plug which determines the concentration profile of the sampling zone [12] and thus affects the reproducibility of the analysis. Moreover, the first two parameters have also an important influence in the sampling rate. Accordingly, it was verified that a flow rate of 4·8 ml/min for the two carrier solutions ($Q_2$ and $Q_3$), a length of 180 cm for $R_1$ and the injection of 80 µl sample volumes provided an adequate dispersion of the sample plug, which arrived at the second valve ($V_2$) with a concentration profile suitable for the cleavage, and enabled a sufficiently wide range of dilutions by using re-sampling times from 14 to 26 s.

The channel $Q_4$ was added to the manifold in order to obtain an additional on-line dilution by increasing the dispersion of the re-sampled portion in its transport toward the detector, and also by creating a detector (AAS and FP) inlet overpressure. This overpressure which results from imposing a flow rate at the entry of the nebulizer that is slightly higher than recommended by the manufacturer reduces the efficiency of the nebulization, decreases the analytical signal and improves the reproducibility of the results [14]. The channel $Q_4$ was also used to add a lanthanum (III) solution that acted as an interference suppresser in the determination of calcium and magnesium.

In the determination of metallic ions in waters, re-sampling times from 10 to 26 s were used which allowed sampling rates from 230 to 90 determinations per hour (table 1). The automatic system permitted the analysis of a wide range of concentration allowing dilution levels up to 10 000-fold. Despite the high dilution levels attained the system showed a good reproducibility with a relative standard deviation (RSD) of less than 3%.

*Table 1. Concentration range determined and re-sampling times used for the determination of metallic ions in waters.*

| | Concentration range* | | Re-sampling time† | | |
|---|---|---|---|---|---|
| Element | Min. | Max. | Min. | Max. | RD (%)‡ |
| Ca | 15 | 58 | 17 | 21 | <1·5 |
| Mg | 4 | 14 | 24 | 26 | <2·5 |
| Ng | 113 | 517 | 20 | 21 | <3·0 |
| K | 4 | 71 | 15 | 17 | <3·0 |
| Fe | 35 | 1732 | 10 | 21 | <2·6 |
| Cu | 29 | 5557 | 17 | 21 | <3·5 |
| Zn | 37 | 4585 | 12 | 16 | <8·7§ |

* Concentration range determined (minimum and maximum value—mg/l).
† Re-sampling time used in the determination (minimum and maximum value—seconds).
‡ Relative deviation of the developed methodology to the reference method.
§ Please see text.

*Table 2. Comparison of the results obtained in the determination of Ca, Mg, Na, K, Fe, Cu and Zn in water samples.*

| | Equation parameters | | | | | |
|---|---|---|---|---|---|---|
| Element | $N$* | $C_0$ | $S$ | $R$† | $t_{0\cdot025}$‡ | $t_{0\cdot025}$§ |
| Ca | 12 | −1·215 | 1·064 | 0·9988 | −1·415 | 2·201 |
| Mg | 12 | 0·2556 | 0·9715 | 0·9969 | 0 | 2·201 |
| Na | 20 | −6·762 | 1·101 | 0·9975 | 1·379 | 2·093 |
| K | 20 | −0·1783 | 1·013 | 0·9986 | −0·6346 | 2·093 |
| Fe | 9 | −2·423 | 1·017 | 0·9999 | −1·418 | 2·306 |
| Cu | 10 | −1·299 | 1·099 | 0·9991 | −2·087 | 2·262 |
| Zn | 13 | −14·36 | 1·024 | 0·9994 | −0·596 | 2·179 |

* Number of samples.
† Correlation coefficient.
‡ Calculated values for a two-tail t-test.
§ Tabulated values (95% confidence level).

The results obtained with the FIA methodology ($C_f$), when compared with those obtained with the reference methods ($C_r$) through the relation $C_f = C_0 + SC_r$ showed a good agreement between both methodologies (table 2) as could be perceived by slopes ($S$) and correlation coefficients ($C_0$) close to unity and relative deviations, expressed in percentage (RD%), of less than 3·5% for all the species except for zinc (table 1). For zinc the average RD% was 2·5%, although one of the samples presented a RD% of 8·7%.

### Conclusions

The results obtained with the automatic dilution system described in this paper for the determination of several metallic cations in water samples of different origins were in a good agreement with results obtained using reference methods. The system could be used as an efficient and flexible dilution procedure both in process control and routine analysis.

The methodology described provides a wide range of dilution factors that are automatically and flexibly adapted to a sample's concentration by simply changing the re-sampling time that controls the rotation of the valves. This re-sampling time selection is done at run-time and does not require an operator. The extent of dilution attained (up to 10 000-fold dilutions) showed that this system could be used for the analysis of high concentration samples. The results obtained in the analysis of metallic cations in water samples, which had a wide and unpredictable concentration range, suggest that the analytical system could be successfully applied to the analysis of other species and other matrices, for example food products or biological fluids.

The implementation of the analytical system in process control or routine laboratories is simple because the equipment is easily available and operation is straightforward. Considering the results obtained with the detectors used in this work (AAS and FP), the system should be simple to adapt to other detectors commonly used in flow analysis, given an aquisition card and a microcomputer.

### Acknowledgements

The authors gratefully acknowledge the financial support from Direcção Geral do Ambiente.

### References

1. Ruzicka, J. and Hansen, E. H., *Analytica Chimica Acta*, **99** (1978), 37.

2. Stewart, K. K. and Rosenfeld, A. G., *Analytical Chemistry*, **54** (1982), 2368.

3. Garn, M. B., Gisin, M., Gross, H., King, P., Schmidt, W. and Thommen, C., *Analytica Chimica Acta*, **207** (1988), 225.

4. Lima, J. L. F. C. and Rangel, A. O. S. S., *American Journal of Enology and Viticulture*, **41** (1990), 284.

5. Lima, J. L. F. C., Rangel, A. O. S. S. and Roque da Silva, M. M. S., *Atomic Spectroscopy*, **12** (1991), 204.

6. Fang, Z., Weiz, B. and Sperling, M., *Analytical Chemistry*, **65** (1993), 1682.

7. Fang, Z., in *Flow Injection Atomic Spectroscopy*, Ed. Burguera, J. L. (Marcel Dekker, New York, 1989), 103.

8. Tyson, J. F., *Spectrochimica Acta Review*, **3** (1991), 169.

9. Luque de Castro, M. D. and Tena, M. T., *Talanta*, **42** (1995), 151.

10. Reis, B. F., Jacintho, A. O., Mortatti, J., Krug, F. J., Zagatto, E. A. G., Bergamin, F. H. and Pessenda, L. C. R., *Analytica Chimica Acta*, **123** (1981), 221.

11. Alegret, S., Alonso, J., Bartroli, J., Machado, A. A. S. C., Lima, J. L. F. C. and Paulis, J. M., *Quimica Analitica*, **6** (1987), 278.

12. Ruzicka, J. and Hansen, E. H., *Flow Injection Analysis*, 2nd edn (John Wiley & Sons, New York, 1988).

13. American Society for Testing and Materials, *Annual Book of ASTM Standards*, (ASTM, Philadelphia, 1979), 31.

14. Lima, J. L. F. C. and Rangel, A. O. S. S., *Food Control*, **7** (1991), 146.

